# Early application of cryoanalgesia to the brachial plexus prevents development of phantom limb pain after traumatic forequarter amputation: A case report

**DOI:** 10.1016/j.tcr.2022.100678

**Published:** 2022-07-13

**Authors:** Lizabeth A. O'Connor, Bryan Houseman, Daniel Taffe, Curtis C. Quinn

**Affiliations:** a Elliot Health System, Division of Thoracic Surgery, 1 Elliot Way, Manchester, NH 03103, United States; b Elliot Health System, Division of Orthopedic Trauma, 1 Elliot Way, Manchester, NH 03103, United States

**Keywords:** Cryoanalgesia, Phantom limb pain, Traumatic amputation, Chronic pain, Neuralgia

## Abstract

**Background:**

Amputation of an extremity frequently results in significant phantom limb pain. The etiology of which is not well understood. Central and peripheral factors appear to play a role. Pain relief interventions often are attempted several weeks to months later. Peripheral nerve injury can rapidly result in cortical somatosensory changes potentially making early intervention important in preventing any permanent changes in nerve pathways.

**Case report:**

We present a case of traumatic forequarter (interscapulothoracic) amputation treated with cryoanalgesia of the brachial plexus for pain control <72 h after injury. The patient denied painful phantom limb pain and postoperative pain at the surgical site immediately following surgery and over a six month follow up period.

**Conclusion:**

Cryoanalgesia facilitates extended duration of pain control of the affected peripheral nerve which may be of particular benefit in patients sustaining either surgical or traumatic amputations, particularly when applied early to prevent the transmission of noxious signals to the central nervous system.

## Introduction

Phantom limb pain is a phenomenon that produces a painful sensation in a traumatic or surgically amputated extremity. Studies have demonstrated a prevalence rate of 50 % to 80 % with varying severity and is more common in the upper extremity compared to lower extremity [1], [2]. Often this pain can be quite debilitating and chronic with no standard approach for managing these symptoms as the true pain-generating mechanism is not fully understood. However, researchers believe the changes occur between the peripheral sensory nerve and the brain due to signals that are misinterpreted or a result of processing errors [3].

Frequently, interventions aimed at alleviating these symptoms are initiated several months after the amputation once patients report intolerable pain and is difficult to treat [4], [5]. Often, interventions such as peripheral nerve injections are performed successfully but are only temporary. The greater duration in time from amputation to intervention may produce a stronger memory of an aberrant nerve pathway resulting in noxious ectopic pain signals potentially due to permanent changes in the sympathetic nervous system [3].

Cryoanalgesia is a technique used to inhibit the propagation of sensory nerve pain signals by freezing the targeted nerve. It results in temporary, but long-term (several months) pain relief with subsequent regeneration and restoration of function [6]. We present a patient with a traumatic upper extremity partial amputation who required surgical completion with intraoperative application of cryoanalgesia to the brachial plexus. Phantom limb pain is thought to result from persistent central nervous changes in response to abnormal peripheral nerve signals transmitted from the severed nerve. Early and long-lasting nerve block achieved with cryoanalgesia may prevent phantom limb pain by inhibiting these signals.

## Case report

**Fig. 1:**
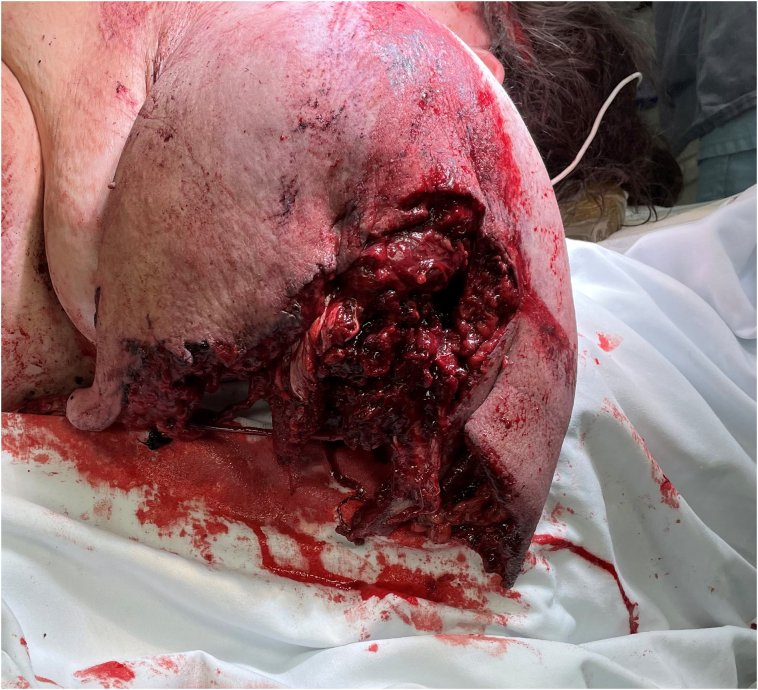
Partial view of the patients left upper extremity traumatic amputation at presentation to the Emergency Department.

A left-hand dominant, 30-year-old female was the unrestrained driver of a highway speed head-on motor vehicle collision. She presented to a Level II trauma center with a traumatic partial amputation (80 %) of the left arm at the proximal humerus [Fig. 1]. She was ejected from the vehicle and upon emergency medical service arrival, was found to be alert and conversant. A tourniquet was placed proximal to the amputation site to control bleeding. Injuries after examination and comprehensive computerized tomography scanning included a traumatic proximal trans-humeral amputation in the presence of a segmental humeral shaft remnant, an open left scapular body fracture, and left scapulothoracic dissociation with an associated acromioclavicular joint separation >3 cm. The patient's left hand had been packaged and accompanied the patient. The remaining intercalary segment of the left upper extremity did not accompany the patient. Additionally, the patient sustained a left pulmonary contusion, left axillary artery transection, 4-cm left ear laceration, and a left talus fracture.

After initial evaluation and resuscitation, a stepwise plan was formulated collaboratively between the orthopedic, thoracic surgery services, and interventional radiology. Potential arterial hemorrhage was felt to be the most immediate concern and coiling of the left subclavian artery was performed by the interventional radiology team. Afterward, the patient underwent wound washout with control of the hemorrhage with suture ligation of the distal subclavian vessels, staged forequarter (interscapulothoracic) amputation, and placement of a vacuum-assisted closure device.

**Fig. 2:**
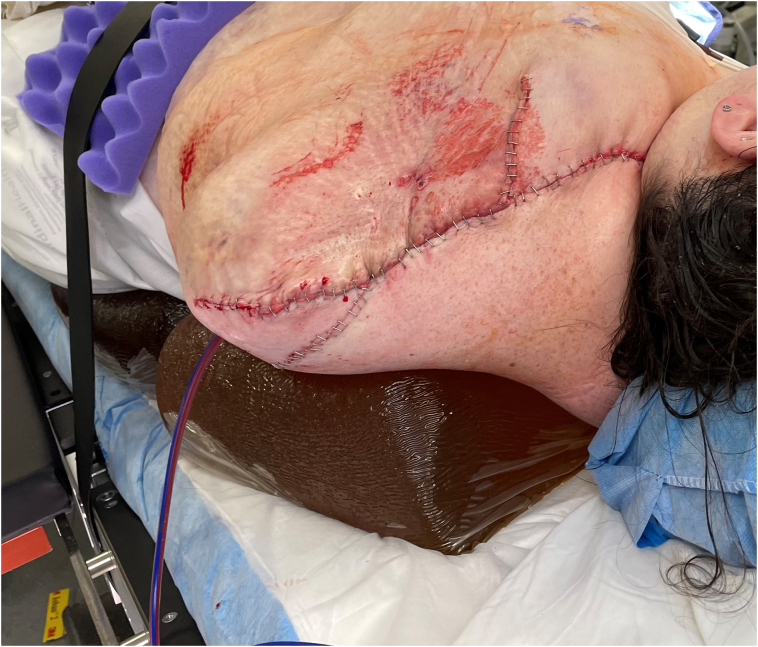
Postoperative view of completion forequarter amputation.

**Fig. 3:**
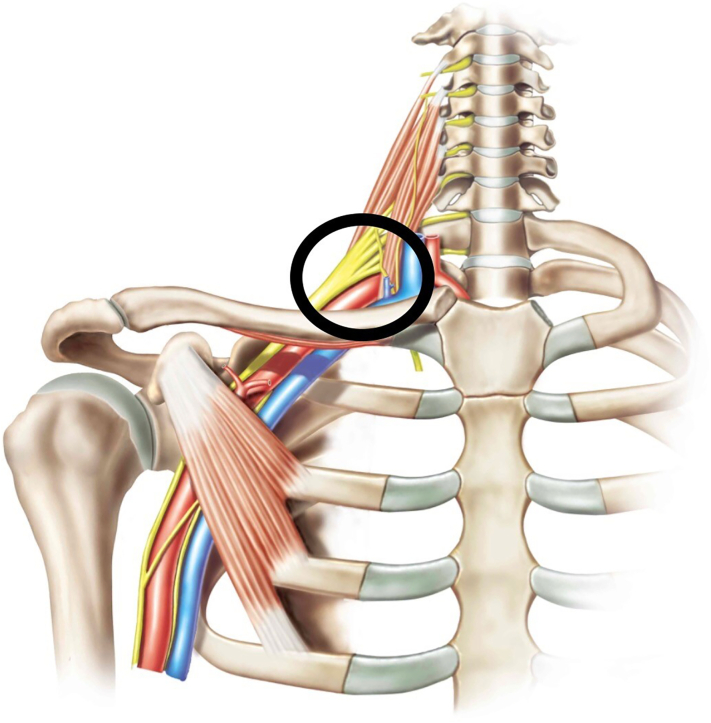
Schematic of brachial plexus anatomy which demonstrates the point of application of cryoanalgesia to include the medial, lateral, and superior cords.

**Fig. 4:**
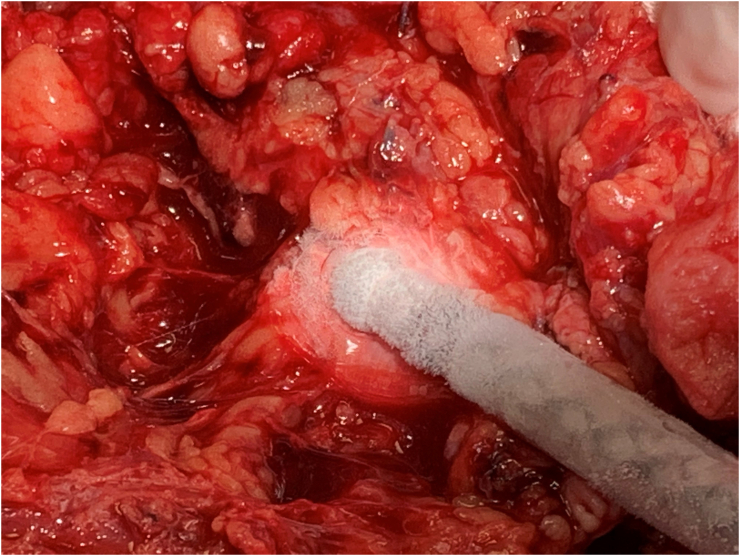
Intraoperative view of the application of cryoanalgesia to the left brachial plexus.

Postoperatively the patient was admitted to the intensive care unit. On postoperative day one, the patient was extubated and transferred to the surgical floor. On hospital day three, the patient was brought back to the operating room and underwent primary closure of the amputation site [Fig. 2]. Prior to wound closure, cryoanalgesia was applied under direct visualization using AtriCure's cryosphere probe (AtriCure, Inc., Mason, OH) to the left brachial plexus, including the medial, lateral, and posterior cords for 120 s at −60 °C [Fig. 3, Fig. 4]. Postoperatively the patient was transferred back to the surgical floor.

The patient was hospitalized for eleven days and subsequently discharged to an acute rehabilitation facility for two weeks before returning to her home. The patient was placed on gabapentin 300 mg every eight hours following her index surgery and remained on this medication during her entire hospitalization. Over the course of the patient's hospitalization, she reported only mild phantom tingling sensation but no pain. While the patient was at the rehabilitation center, medical staff changed this medication to pregabalin 50 mg twice a day due to increasing reports of fatigue and occasional phantom pain sensations. The patient described this increased sensation as not debilitating or particularly bothersome. She was discharged from the rehabilitation center on the same medication and dose. Additionally, she received medication for muscle spasms remote to the amputated extremity (cyclobenzaprine 5 mg three times a day for two weeks after surgery) and pain associated with her ankle fracture (oxycodone 5 mg every six hours as needed). Outpatient follow up occurred at two, four, six, twelve, and twenty-four weeks with pain scores measured on an 11-point numerical rating scale of 0, 2, 0, 0, and 0 respectively. At her 12-week postoperative visit, she denied phantom limb pain or new paresthesias and was no longer requiring any opioids for her ankle pain. During her six-month evaluation, she remained off opioids and reported no phantom limb pain symptoms or paresthesias.

## Discussion

The etiology for the development of phantom limb pain is not clear and several theories exist including cortical reorganization that solidifies painful nerve memories and pathways [1], [2]. Peripheral nerves may develop a neuroma over time contributing to painful sensations and continue to supply negative input to the central nervous system (CNS). Interventions aimed to decrease painful peripheral nerve input to the CNS could mitigate the generation of phantom limb pain [1]. Unfortunately, treatment for phantom limb pain tends to occur once the symptoms have been established and patients whom no longer can tolerate it, seek treatment [6]. The patient presented herein received early intervention soon after amputation of the affected extremity. This resulted in minimal post-amputation pain and no evidence of phantom limb pain.

Cryoanalgesia has been used since the 1960s for freezing of peripheral nerves to treat pain and this technology has improved with clarified application settings related to optimal temperature and duration of treatment [7], [8]. It is a peripheral nerve blocking technique resulting in extended pain control for approximately two to three months. Through the use of pressurized nitrous oxide, cold temperatures are produced which injure the axon and myelin sheath, resulting in Sunderland Stage II nerve injury [7], [8]. This results in the temporary inability of a peripheral sensory nerve to conduct a pain signal. Since the structural elements of the nerve bundle (endoneurium, perineurium, and epineurium) are preserved, nerve regeneration will occur at a rate of 1–2 mm per day with the recovery of nerve function. Using the same standardized settings, cryoanalgesias use has increasingly been employed as an adjunct for opioid-sparing pain management within thoracic [9], pediatric [10], and orthopedic [11], [12] surgery. To date, there are no published studies examining the application of cryoanalgesia for pain control and prevention of phantom limb pain applied at the time of amputation. Recently, a study has been registered to assess the role of cryoanalgesia in the treatment of post-amputation phantom limb pain present at least 12 weeks after amputation [13].

The impact of cryoanalgesia for patients suffering from phantom limb pain is an important option to consider given the frequent use of opioids as first-line treatment for achieving pain control. In an effort to minimize patients suffering, the potential to develop chronic pain, and contribution to the opioid epidemic, cryoanalgesia should be considered a viable option as a pre-emptive pain control intervention. When applied early in the course of treatment, it may mitigate potential long-term intractable and debilitating pain associated with phantom limb pain. The use of gabapentinoids in conjunction with the application of cryoanalgesia may produce a synergistic effect. This relationship and overall efficacy of cryoanalgesia for the treatment of phantom limb pain requires future research with prospective randomized studies.

## Statement of informed consent

Informed consent was obtained from the patient included in the study.

## Funding

This research did not receive any specific grant from funding agencies in the public, commercial, or not-for-profit sectors.

## Declaration of competing interest

L. O'Connor and C. Quinn serve as educational faculty to AtriCure®. B. Houseman and D. Taffe declare that they have no conflict of interest, nor financial and personal relationship with other organizations that could inappropriately influence the work.

The authors received no financial support for the research, authorship, and/or publication of this article.
